# Treatment of Muco-Membranous Colitis
1Dr. de Langenhagen: The Lancet, April 30, 1904.


**Published:** 1904-06-18

**Authors:** 


### TREATMENT OF MUCO-MEMBRANOUS COLITIS.[1]

Muco-membranous Colitis—a chronic, non-parasitic, catarrhal inflammation of the large bowel—occurs more especially in those of gouty and neurotic habit. In some cases the arthritic element preponderates, and with this the colitis is usually less severe and more easily curable; in others the neurotic influence is extreme and the symptoms are more obstinate. The characteristic evidences of the disease are those of intestinal atony associated with secretion of mucoid or membranous matter, which may be evacuated in the form of shreds, membranes, etc. The atony involves constipation and this ultimately leads to mechanical irritation and hypersecretion; hence there may be diarrhœa at times. Pain is a third fundamental element of the disease. This may exist as a sense of weight and discomfort in the iliac fossa coming on some hours after meals, or it may occur in severe paroxysms, apt to be mistaken for hepatic colic or intestinal obstruction. There is on examination a general flabbiness of the abdominal wall, and parts of the large bowel, especially the cæcum, may be felt to be distended and atonic, whilst the sigmoid flexure is usually contracted. Dyspeptic disturbances, enteroptoses, and sand in the fæces (intestinal lithiasis) may also be noted together with loss of flesh and various neuropathic phenomena. The indications for treatment are to select an appropriate diet, to secure evacuation of the contents of the atonic intestine, to overcome the intestinal catarrh, and to improve the underlying diathesis. The diet must be nourishing and must not leave an abundant or irritating residuum in the intestine—for example, milk, meat soups, boiled white fish, minced lean meats, mashed potatoes may be permitted, but alcoholic drinks, fatty foods, green vegetables, and raw fruits must be denied. Bread, if stale or toasted, is allowed. The best beverage is spring water. In obstinate cases even greater strictness must be practised. Irrigation of the large bowel is an important factor in successful treatment. Its object is not mere removal of the contents of the rectum, but to cleanse and improve the intestinal mucous membrane. The water employed for this purpose should have a temperature of 35° to 45° C. and may be medicated by the addition of a demulcent, as marshmallow or linseed, or a mild antiseptic as boric acid, and a quart or more of fluid should be introduced slowly and under low pressure by means of a fountain-injector. At first the patient should be lying on his back and should then gradually turn on to his right side so as to promote the access of the fluid to all parts of the colon. The irrigation may be repeated daily or less frequently according to the severity of the case. Mild laxatives, abdominal massage and electrical applications all have their sphere of usefulness in overcoming intestinal atony. The most suitable health resorts for the treatment of colitis are Plombières and Châtelguyon. The former possesses springs which claim to be anti-rheumatic, sedative to the nervous system, and capable of reducing local congestion. In addition, special apparatus and methods have been developed for the practice of intestinal irrigation, and the same may be said of the general hygienic appointments. Drugs for the most part have but an unimportant position in the treatment of colitis. Belladonna suppositories or ointment or poultices to the abdomen may be needed to relieve pain and simple remedies may be necessary to check dyspeptic troubles. But these latter, as well as other symptoms, will disappear as the condition of the intestine is improved.

[1] Dr. de Langenhagen: The Lancet, April 30, 1904.

